# Predation affects the evolution of sex-specific longevity

**DOI:** 10.1098/rsbl.2024.0451

**Published:** 2024-11-13

**Authors:** Tanya M. Pennell, Masako Katsuki, C. Ruth Archer, Manmohan D. Sharma, Kensuke Okada, David J. Hosken

**Affiliations:** ^1^ Centre for Ecology & Conservation, Faculty of Environment, Science and Economy (ESE), University of Exeter, Cornwall Campus, Penryn, UK; ^2^ Department of Agricultural and Environmental Biology, The University of Tokyo, Yayoi 1-1-1, Bunkyo-ku, Tokyo 113-8657, Japan; ^3^ Institute of Evolutionary Ecology and Conservation Genomics, University of Ulm, Ulm, Germany; ^4^ Laboratory of Evolutionary Ecology, Faculty of Environmental, Life, Natural Science and Technology, Okayama University, Okayama, Japan

**Keywords:** experimental evolution, individual quality, natural selection, sexual selection, sexual conflict

## Abstract

Predation, a major cause of natural selection, is classically thought to target the weak and sick. However, predators can target animals with condition-dependent sexual traits, and therefore, high-quality individuals can also be the focus of predation. Thus, it is not always clear which individuals are the foci of predators or how this affects trait evolution. Here, we tested for evolutionary effects of sex-specific predation on male and female longevity using replicate populations of the broad-horned flour beetle *Gnatocerus cornutus*. We found that male-limited predation resulted in the evolution of reduced male and increased female longevity, while female-limited predation had no effects on the longevity of either sex. We also document the costs of reproduction. Coupled with other findings, our results suggest that predation impacts high-quality males and, because of negative intersexual genetic correlations, this increases female longevity.

## 1. Introduction

Predation is a major mechanism of natural selection, with impacts on prey behaviour and morphology (e.g. [1–8]), as well as ageing and other life-history components (e.g. [9–11] and see [12]). Overall, predation can be a strong source of selection, but it is not clear whether this selection primarily targets high- or low-quality individuals. That is, are individuals that would otherwise live longer and reproduce more the targets of predation, or are the sick and weak targeted?

Classically, predation was thought to target low-quality individuals, such as those in poor condition, the injured, or those carrying greater parasite loads [13,14], with evidence to support this (e.g. [13,15–21]). Thus, low-quality individuals may be especially susceptible to predators because they are less able to evade them. Predation on low-quality individuals could have positive effects on population growth by removing low-fitness genotypes or, in some cases, by limiting the spread of disease [22,23].

High-quality individuals can also be at greater predation risk if, for example, predators target larger individuals [24,25]. Perhaps larger prey are more conspicuous, or predators optimally forage [26–28], with larger animals representing higher value food. Predation based on quality may also be sex-specific, with, for example, highly fecund, large females targeted because of their size or foraging activities (e.g. [29,30]). Predation can also impact high-quality males. In some taxa, males signal their quality to rivals and females via elaborate sexually selected traits, such as mating calls, weapons and vibrant pigmentation (e.g. [31,32]). These traits are usually honest advertisements of male quality [33–35]—greater trait expression, therefore, signals higher quality. However, increased sexually selected trait expression can increase male conspicuousness, and so, predators may target high-quality males (e.g. [1,36–38]). Overall, selective predation of high-quality individuals may negatively impact population growth by removing alleles conferring high reproductive rates. Moreover, selection against sexually selected traits by predators can affect mate choice, male–male competition, and even speciation through sexual selection [39].

To fully understand links between predation, individual quality and trait evolution, it is useful to explore how predation affects males and females separately. This is particularly true when traits have a shared genetic architecture across the sexes [40], as selection on one sex can have knock-on effects on phenotypes in the opposite sex (e.g. [41]).

Here, we tested how sex-specific predation affects longevity in the broad-horned flour beetle *Gnatocerus cornutus*. Longevity is a key fitness trait and is often used as a general marker of individual quality (reviewed in [42]). Previously, we used eight generations of experimental evolution to explore the impacts of sex-specific predation on male trait evolution [43]. This indicated that predacious assassin bugs (*Amphibolus venator*) directly or indirectly target males with large mandibles, a sexually selected trait found in males but not females [43]. This led to the evolution of smaller mandibles in populations evolving under male predation [43]. However, it is unclear how this affected other key fitness traits such as lifespan. As males with smaller mandibles are of lower sexual quality [43], we also expect lifespan to be reduced in these males, although trade-offs between lifespan and other fitness components make precise predictions difficult [44]. Moreover, because lifespan may be genetically correlated across the sexes (e.g. [45,46]), any changes in male longevity may have correlated impacts on female lifespan. Here, we extend previous work [43] to test how predation affects sex-specific longevity and test for costs of reproduction, as both males and females may incur costs due to energetic demands of sexual interactions or ejaculate/egg production [47,48]. In doing so, we test novel predictions while also retesting previous findings in these populations—this is important given that reproducibility is a significant research concern and studies replicating previous work are rare [49,50].

## 2. Material and methods

### (a) Prey stock

The *G. cornutus* beetle culture originated from adults collected in Miyazaki City (Japan) and has been maintained in the laboratory of the National Food Research Institute (Japan) for approximately 50 years [43].

### (b) Predator stock

The assassin bug, *A. venator*, is a predator of stored-product insect pests including flour beetles [51–53] (see electronic supplementary material for stock details).

### (c) Experimental evolution under sex-specific predation

**Figure 1.**
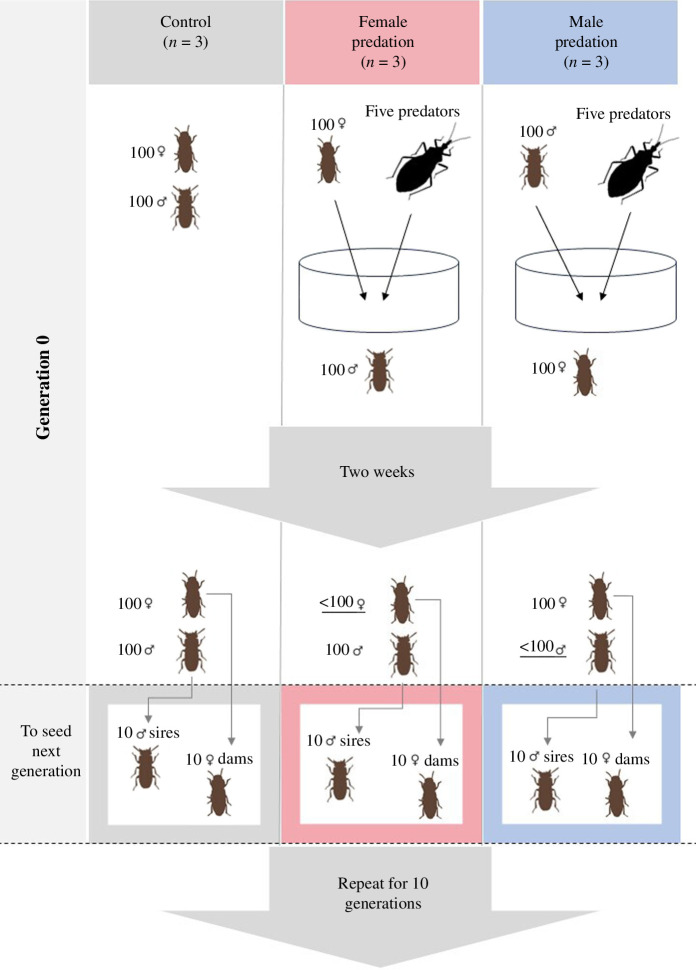
Diagram of the experimental evolution protocol. Protocol for the three replicates of each treatment (control = no predation, female-limited predation and male-limited predation).

We collected 900 male and 900 female *G. cornutus* from the stock culture and haphazardly generated nine groups of 100 males and 100 females to establish three male-predation populations, three female-predation populations and three control (no predation) populations (generation 0; see figure 1). In the predation populations, 100 males (or females) were housed in a plastic container (150 mm diameter and 50 mm high) containing excess beetle food (45 g). Five adult female *A. venator* (20–35 days old) were then randomly collected from the predator culture and placed into the container, and the males (male-predation population) or females (female-predation population) were exposed to them for two weeks. We then selected 10 of the males (females) that survived the two weeks to act as sires (dams) of the predation treatments—10 opposite-sex individuals that were not exposed to predation in each population were also haphazardly allocated as the non-selected dams (sires). Survival during this predation protocol was approximately 20% in both sexes. To propagate control populations, 10 males and 10 females were haphazardly selected per population to act as sires and dams. For each population or treatment, these 10 males and females were placed in a plastic cup (diameter 95 mm and height 50 mm) with 70 g of medium for two months, with males able to mate and females to lay eggs, until final instar larvae were obtained and collected to obtain adults for subsequent generations. When adults reached 14 days old, 100 males and 100 females per population were randomly selected to (potentially) seed the next generation and, in the predation treatments, exposed to predators as above. We then selected surviving animals as above and repeated for 10 generations (figure 1).

### (d) Retesting previous findings

Male fighting ability, mandible size and body size, and female body size and lifetime reproductive success (LRS), were previously measured in the same experimental populations we use here [43]. We first tested whether we could replicate previous findings by measuring the same traits in later generations. At generation 9, we haphazardly collected 20 males per population for measurement of fighting ability (*n* = 180) in virgin males. Males were scored as winners or losers (binary data) depending on fight outcomes (see electronic supplementary material for protocols for measurement of all traits). Additionally, 40 males and 40 females per population (360 animals per sex) were haphazardly collected and divided into two groups (20 males and 20 females per group per population). One group (*n* = 180 per sex) was used for measuring male traits (mandible and body size) and female traits (body size and LRS) in mated individuals. The second group was used for the measurement of the body size of virgin beetles (*n* = 180 per sex). At generation 10, 20 males and 20 females per population were haphazardly collected for measurement of male traits (mandible size and body size) and a female trait (body size; *n* = 180 per sex) in mated individuals.

### (e) Predation and the evolution of longevity

In addition to replicating tests of previous findings [43], we measured longevity in males and females. The beetles collected at generation 9 (see above; virgin and mated) were also used for measurement of longevity, as were the mated individuals collected at generation 10 (see above). Due to possible links between early development and adult longevity, we also haphazardly collected males and females (10 mating pairs per population) to assay the egg-adult development time of their offspring (40 eggs from 10 pairs; *n* = 175 females; *n* = 163 males).

### (f) Statistical analyses

All analyses were carried out in R software (v. 4.3.1) [54]. For each trait, we ran a linear mixed effect model (lmer), using data collected from mated individuals at generations 9 and 10 (package ‘lme4’ [55]). Population trait mean was the response variable, experimental treatment was a fixed effect, and generation was a random effect. We also ran separate linear models (lm in base R) for trait measurements taken from virgin individuals at generation 9, excluding the random effect of generation, as virgin individuals were not measured at generation 10. Population rates of winning were used as the response variable for the analysis of fighting ability and were logit transformed prior to analysis. Population means were used in all analyses because population is the biologically relevant unit of replication in an experimental evolution study [56]. The significance of experimental treatment (where *p* < 0.05) was assessed using either the ‘step’ function in package ‘lmerTest’ [57] (for lmer) or ‘drop1’ in base R (for lm). Where significant, *post hoc* analyses were carried out using pairwise comparisons (package ‘lsmeans’ [58] for lmer or package ‘emmeans’ [59] for lm). Analyses were carried out separately for male and female traits at each generation. While population is the unit of replication in an experimental evolution study, Shapiro–Wilk’s tests were nonetheless performed to assess the skew of data within each population. In cases of non-normality, we also conducted either lmer or lm models using population median values. For binary data (fighting outcomes), median values are not appropriate.

Additionally, paired *t*-tests were used to compare the mean longevity of virgin and mated individuals at generation 9. Where appropriate, median (rather than mean) values were also used in analyses.

## 3. Results

**Figure 2.**
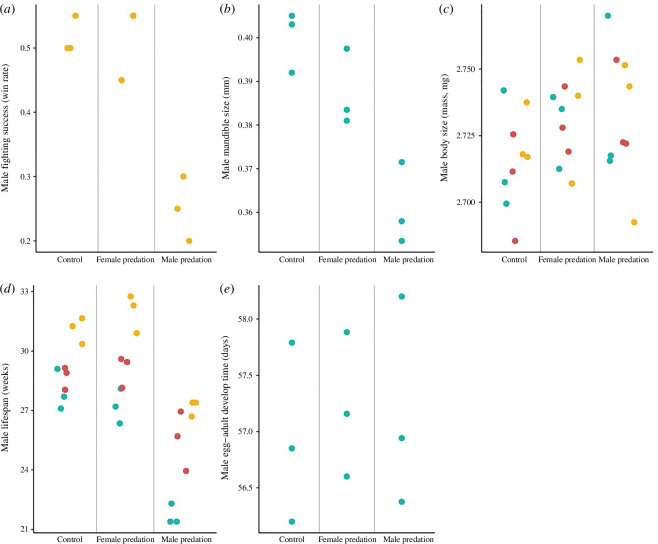
Male trait values after 9 and 10 generations of experimental evolution. Points represent mean population values (three per treatment) for each male trait: (*a*) fighting success; (*b*) mandible size; (*c*) body size; (*d*) longevity; (*e*) egg–adult development time. Values derived from virgin males at generation 9 are represented by yellow points. Values derived from mated males at generations 9 and 10 are represented by turquoise and red points, respectively. Development time refers to the offspring of generation 9 individuals (see electronic supplementary material, figures S1–S5, for distributions of raw data).

**Figure 3.**
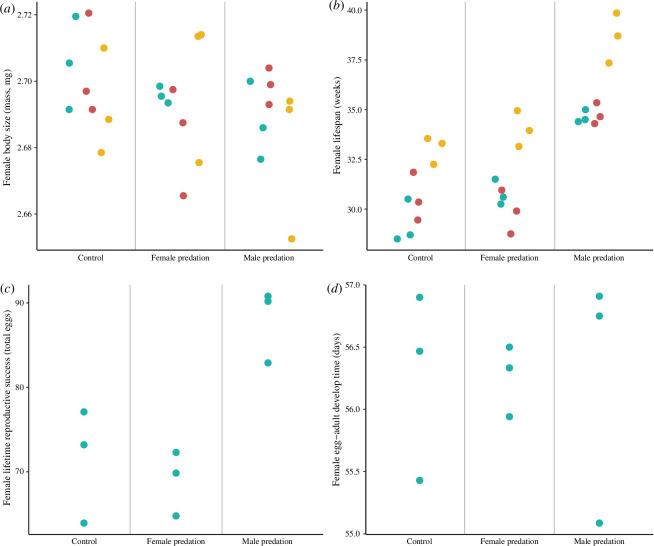
Female trait values after 9 and 10 generations of experimental evolution. Points represent mean population values (three per treatment) for each female trait: (*a*) body size; (*b*) longevity; (*c*) LRS; (*d*) egg–adult development time. Values derived from mated females at generations 9 and 10 are represented by turquoise and red points, respectively. Values derived from virgin females at generation 9 are represented by yellow points. Development time refers to the offspring of generation 9 individuals (see electronic supplementary material, figures S6–S9, for distributions of raw data).

As found previously [43], and still apparent here after further experimental evolution, body size was unaffected by predation (generations 9 and 10; mated males: figure 2*c*, *F*_2,14_ = 2.27, *p* = 0.138; mated females: figure 3*a*, *F*_2,14_ = 2.40, *p* = 0.125). Similar body size results were also found in virgin males and females at generation 9 (see electronic supplementary material, table S1, for all results).

Findings for male mandible size (mated males) and fighting ability (virgin males) at generation 9 were also in agreement with previous work [43]. We observed a significant difference in male mandible size between treatments (figure 2*b*; *F*_2,6_= 16.51; *p* = 0.004). *Post hoc* analyses revealed that males evolved smaller mandibles under male-limited predation (figure 2*b*; all *p* < 0.05). Males from the female-predation and control treatments did not differ in mandible size (figure 2*b*; *p* = 0.239). Male fighting ability also differed significantly between treatments (figure 2*a*; *F*_2,6_ = 29.75; *p* < 0.001), with *post hoc* analyses revealing that males evolving under male-limited predation were poorer fighters (figure 2*a*; all *p* < 0.01). Males from the female-predation and control treatments did not differ in fighting ability (figure 2*a*; *p* = 1.0).

Consistent with previous work [43], we also found a significant difference in female LRS between treatments at generation 9 (figure 3*c*; *F*_2,6_ = 12.07; *p* = 0.008), with *post hoc* analyses revealing that females evolving under male-limited predation had higher LRS (figure 3*c*; control–male predation: *p* = 0.018; female predation–male predation: *p* = 0.01). Female LRS from the female-predation and control treatments did not differ (figure 3*c*; *p* = 0.836).

Male longevity differed significantly between treatments (generations 9 and 10; mated males; figure 2*d*; *F*_2,14_ = 33.16; *p* < 0.001); males evolved shorter lifespans in the male-predation treatment compared to the other treatments (figure 2*d*; all *p* < 0.001), which did not differ in male longevity (figure 2*d*; *p* = 0.954). The same findings were obtained at generation 9 in virgin males (see electronic supplementary material, table S1). Additionally, we found that virgin males had significantly longer lifespans compared with mated males in both the female-predation and male-predation treatments (the same trend was non-significant in the control treatment; see figure 2*d* and electronic supplementary material, table S2).

There was a significant difference in mated female longevity between treatments (generations 9 and 10; figure 3*b*; *F*_2,14_ = 47.93; *p* ≤ 0.001); *post hoc* analyses revealed that females lived longer in the male-predation treatment (figure 3*b*; all *p* < 0.001). However, mated females from the female-predation and control treatments did not differ in longevity (figure 3*b*; *p* = 0.711). Similarly, in virgin females, there was a significant difference in longevity between treatments at generation 9 (figure 3*b*; *F*_2,6_ = 28.18; *p* ≤ 0.001), with *post hoc* analyses again revealing that females evolved longer lifespans in the male-predation treatment (figure 3*b*; all *p* < 0.01). Virgin females from the female-predation and control treatments did not differ in longevity (figure 3*b*; *p* = 0.478). As both virgin and mated females had increased longevity in the male-predation treatment compared to other treatments, increases in lifespan associated with male predation cannot be attributed to reductions in mating costs *per se*. Finally, we also found that virgin females had significantly longer lifespans compared to mated females in all treatments (see figure 3*b* and electronic supplementary material, table S2). For the control treatment, this trend was non-significant when median values were used (electronic supplementary material, table S2).

Results remain qualitatively unchanged in other analyses where population medians were used (see electronic supplementary material, tables S1 and S2). We also note that egg–adult development time was not impacted by predation treatment for either males (figure 2*e*; *F*_2,6_ = 0.10; *p* = 0.909) or females (figure 3*d*; *F*_2,6_ = 0.0004; *p* = 1.0; see electronic supplementary material, table S1).

## 4. Discussion

Male predation resulted in the evolution of shorter male but longer female lifespans, while female-limited predation did not affect longevity in either sex. In addition to these novel results, we found that virgin females and males lived longer than mated individuals. Egg-to-adult development time was unaffected by predation in either sex. We also recapitulated findings from an earlier study [43]—specifically, predation impacted males with large mandibles, leading to the evolution of males with smaller mandibles who were less successful fighters. Female LRS also evolved to be greater in the male-limited predation treatment, but the body size of both sexes was unaffected by predation. These results are broadly consistent with other studies demonstrating that predation can affect the evolution of a range of prey phenotypes (e.g. [3–11]). In contrast, female-limited predation had no effect on the evolution of any traits we assessed.

Our finding that male-limited predation led to the evolution of smaller sexually selected mandibles replicates previous findings in this species [43] and is consistent with results from a range of taxa showing that individuals with sexually selected traits can be targeted by predators [31,32] (e.g. [36–38,60–62]). It is possible that males with large mandibles are more visible, increasing susceptibility to predation. Alternatively, mandible size in *G. cornutus* is negatively phenotypically and genetically correlated with male locomotion [63], and locomotion is a predator escape mechanism in flour beetles [64]. Therefore, males with larger mandibles move more slowly, which likely contributes to their increased susceptibility to predation. In this case, locomotion may be the direct target of predation. Males with large mandibles are also better fighters and have higher mating success [65–68], and as with previous work [43], we found that male-limited predation resulted in reduced male fighting ability.

One of our novel findings was that males evolved shorter lifespans when predation was male-limited (female-limited predation had no effect). Lifespan is likely to be highly polygenic and can evolve in response to selection on other traits due to genetic correlations between them (e.g. [69,70]). Here, it appears that locomotion could be correlated with mandible size and longevity (see below). Previous findings have linked mandible size and sexual-fitness components [65,67,68,71], and our longevity result suggests that male-limited predation also results in the evolution of reduced non-sexual quality in males. This reduction in general male quality is consistent with a reduction in mandible size because sexually selected traits are usually indicators of male condition [33–35]. Moreover, when condition is manipulated through diet, poor condition (low quality) males have small mandibles and reduced lifespans [72].

Our other major finding was that the impact of male-limited predation extended to females, with female longevity increasing under the male-predation treatment (but not in the female-predation treatment). This finding suggests a negative intersexual genetic correlation between male and female longevity [40,73]. This is consistent with previous work and our recapitulated results, showing that male-limited predation resulted in a correlated increase in female LRS [43]. A negative intersexual correlation for longevity may arise if the same genes that cause a reduction in male lifespan cause resources to be directed in entirely different ways in females, leading to an increase in female lifespan. All of this depends on the genetic architecture underlying the covariance of fitness components within and between the sexes [74,75], as well as trade-offs between lifespan and other fitness components, which require further unpacking. These effects may be mediated by juvenile hormone, titres of which correlate positively with male mandible size but negatively with abdomen size in *G. cornutus* [76]. Juvenile hormone also affects longevity in insects (e.g. [77,78]), and its effects on phenotypes are often sex-specific (e.g. [79–81]). Finally, we cannot rule out the possibility that the offspring of males exposed to predators were affected by non-genetic parental effects [82–85]. For example, cues passed from fathers to offspring may affect gene expression and development in offspring, which could partially explain sex-specific effects on lifespan.

We also note that in previous studies, where selection was applied directly on mandible size, there was no evolutionary response in male or female longevity [71,86]. However, this artificial selection directly targeted mandible size, while predation is probably targeting locomotion speed, suggesting that longevity and mandible size are both genetically correlated with locomotion but not each other. As above, further analyses of the genetic variance–covariance matrix are required to test this.

Overall, our results add to a picture of predation on males increasing female quality via shared genetic architecture [43,87]. Consistent with this inference, when quality is manipulated by diet, high-condition (high-quality) females have longer lifespans [72]. Additionally, fitness—the best measure of individual quality—is negatively correlated between the sexes in this species [71], showing that more feminized phenotypes are good for females but bad for males (a hallmark of intralocus sexual conflict [88–90]). The overall lack of evolution resulting from female-limited predation suggests that predation on females was not selective with respect to any traits we assessed.

We also found that females suffered a general cost to mating, since virgins lived longer than mated females. This cost could be due to direct interaction with males or costs of egg production [91–93]. Mated males also suffered from reduced lifespans compared to virgins, which could result from energy expenditure associated with mating or ejaculate production [94]. Causes of longevity costs of reproduction are varied and have been widely documented (e.g. [46,95–97]). Perhaps increased female longevity under male-limited predation was due to reductions in mating costs, which could occur because more aggressive males have larger mandibles, and these males were removed by predation [98]. However, misdirected aggression toward females is uncommon in males of the age tested in our study [99]. Moreover, virgin females (who were not exposed to males as adults) also had longer lifespans in the male-predation treatment (figure 3), suggesting that longer female lifespans associated with male predation are not only caused by reductions in mating costs.

Overall, we showed that female-limited predation generated no micro-evolutionary responses in either sex for the traits we assessed, but male-limited predation reduced male lifespan and increased female lifespan. Thus, predation on males reduces male sexual [43] and non-sexual quality, but also increases female quality [43]. Our findings indicate how predation might impact sex-specific life histories and, in turn, population demography—a topic that is poorly understood [100]. Future work exploring the effects of predation on net population fitness will help unpack this further as will explicit investigation of intersexual genetic covariances.

## Data Availability

Data are available from the Dryad Digital Repository [101]. Some methods are available as electronic supplementary material [102].
